# Contemporary Oral and Dental Health Care: Issues and Challenges

**DOI:** 10.3390/healthcare14183100

**Published:** 2026-09-20

**Authors:** Mauro Henrique Nogueira Guimarães de Abreu

**Affiliations:** 1 Department of Community and Preventive Dentistry, Universidade Federal de Minas Gerais, Belo Horizonte 31270-901, MG, Brazil; maurohenrique@ufmg.br; Tel.: +55-31-34092434; 2 Department of Health Policy & Health Services Research, Henry M.Goldman School of Dental Medicine, Boston University, Boston, MA 02118, USA; mauro@bu.edu

Oral and dental health care, when organized in different countries of the world, has one main objective—to improve oral health among individuals and populations. This statement seems to be simple or naïve, but in reality, this is more complex and involves a diverse network of challenges, from structural factors to behavioral and biological dimensions. First of all, we need to clearly define what oral health is. According to the FDI World Dental Federation, this is “a multifaceted concept involving the ability to speak, smile, smell, taste, touch, chew, swallow, and express a full range of emotions through facial expressions with confidence and without pain, discomfort, or disease of the craniofacial complex” [1]. Classical and very well-documented evidence has shown that oral diseases are quite frequent in the global population [2] and impact oral health-related quality of life [3]. To face this burden of oral disease, we must first analyze the concept of health promotion in the 1980s. The Charter of Ottawa pointed out that the basic conditions for health are peace, shelter, education, food, income, a stable ecosystem, sustainable resources, social justice, and equity [4]. Hence, there is a challenge for oral and dental care services to assume that actions to promote health are frequently beyond dental/oral clinical treatment. In this Special Issue, we have published 10 manuscripts from different countries, continents and themes. All manuscripts have rigorous methods and interesting results covering an umbrella of topics. The published manuscripts involved the description and analyses of oral conditions, preventive care in Dentistry, factors that affected the quality of care, including the quality of life of patients and dental team.

Nowadays, health services research has a rich opportunity to advocate for and mediate actions through health promotion as presented by the Charter of Ottawa. We would like to give some examples of these actions. Firstly, extreme situations such as war [5] and climate change [6] are unfortunately more common in recent years. Oral health services must take into account their impact on oral diseases as well as on the management of these services. Another recent challenge for our day-to-day life involves the increased and intense use of technology by our society, including the rapid expansion of social media platforms. The latter has created unprecedented channels for the circulation of misleading content. Hence, misinformation in health has emerged as a serious issue, including in the oral health field [7]. Oral health services should be an institution for disseminating good, clear, and scientifically based information to promote health. Finally, the lack of political power for global oral health could influence the international neglect of the inclusion of oral health care services in primary health care. The integration of the agendas of international organizations, such as WHO, FDI, and IADR, among others, could be beneficial for the real incorporation of oral and dental care services into primary health care [8].

These are only a few examples to inspire more robust research development and also more public health actions to overcome some challenges in oral and dental health care. The decrease in the burden of oral diseases in a healthier world environment, together with the development of more inclusive and sustainable oral and dental health care services, could guarantee a better and happier life for individuals and populations.

## Abbreviations

The following abbreviations are used in this manuscript:

FDI	World Dental Federation
WHO	World Health Organization
IADR	International Association for Dental, Oral, and Craniofacial Research
CNPq	National Council for Scientific and Technological Development
